# Advances in Integrated Multi-omics Analysis for Drug-Target Identification

**DOI:** 10.3390/biom14060692

**Published:** 2024-06-14

**Authors:** Peiling Du, Rui Fan, Nana Zhang, Chenyuan Wu, Yingqian Zhang

**Affiliations:** 1 School of Pharmacy, Hangzhou Normal University, Hangzhou 311121, China; 2023112025016@stu.hznu.edu.cn (P.D.); 2022112025095@stu.hznu.edu.cn (R.F.); 2022112025031@stu.hznu.edu.cn (N.Z.); 2021112012237@stu.hznu.edu.cn (C.W.); 2 Key Laboratory of Elemene Class Anti-Cancer Chinese Medicines, Engineering Laboratory of Development and Application of Traditional Chinese Medicines, Collaborative Innovation Center of Traditional Chinese Medicines of Zhejiang Province, Hangzhou Normal University, Hangzhou 311121, China

**Keywords:** drug target, multi-omics, proteogenomics, single-cell multi-omics, spatial multi-omics

## Abstract

As an essential component of modern drug discovery, the role of drug-target identification is growing increasingly prominent. Additionally, single-omics technologies have been widely utilized in the process of discovering drug targets. However, it is difficult for any single-omics level to clearly expound the causal connection between drugs and how they give rise to the emergence of complex phenotypes. With the progress of large-scale sequencing and the development of high-throughput technologies, the tendency in drug-target identification has shifted towards integrated multi-omics techniques, gradually replacing traditional single-omics techniques. Herein, this review centers on the recent advancements in the domain of integrated multi-omics techniques for target identification, highlights the common multi-omics analysis strategies, briefly summarizes the selection of multi-omics analysis tools, and explores the challenges of existing multi-omics analyses, as well as the applications of multi-omics technology in drug-target identification.

## 1. Introduction

In the field of drug development, identifying and validating drug targets is a crucial and challenging aspect. The success of drug development largely depends on two main factors: the efficacy and safety of the drugs. Studying drug targets not only reveals the active ingredients of traditional Chinese medicine and therapeutic targets within organisms but also helps elucidate the mechanisms of drug action and assists in detecting adverse drug reactions. Specifically, drugs exert therapeutic effects by interacting specifically with key molecules or cellular structures. These drug targets encompass a range of biomolecules, including proteins, enzymes, cell-membrane receptors, DNA or RNA sequences, and key regulatory factors within cellular signaling pathways or biological processes [1].The accurate determination of drug targets enables the purposeful design of a series of drugs that have superior therapeutic effects, safety, and applicability. These drugs typically act more precisely on specific parts or mechanisms, thereby demonstrating remarkable curative effects and bringing significant breakthroughs in disease treatment [2,3,4,5]. In the early stages of drug development, target identification primarily relies on biochemical and molecular biology methods, but these methods are often inefficient and time-consuming. With the emergence of high-throughput technologies, machine-learning computational methods have become an efficient approach for target screening. By leveraging vast amounts of biological data, including molecular structures, biological activities, and genomic information, machine-learning models establish mathematical frameworks to predict interactions between compounds and targets, thus altering the landscape of drug discovery [6,7]. Furthermore, with the advancement of omics technologies, it has significantly enhanced drug-target identification [8,9]. Genomic technologies assist in identifying gene mutations associated with diseases, while transcriptomic analysis reveals gene expression patterns and proteomic research elucidates changes in specific proteins under pathological conditions. Metabolomics can provide the most direct evidence for understanding the physiological and pathological processes of disease metabolism and molecular mechanisms. In this review, we start with the common multi-omics co-analysis strategies and introduce the analysis methods commonly used for drug-target identification, including the integration of transcriptome and proteomics, the integration of transcriptome and metabolomics, and the integration of proteomics and metabolomics. Second, we comprehensively summarize the latest advancements in multi-omics technologies for drug-target identification, such as single-cell multi-omics and spatial multi-omics. Additionally, we review commonly used tools and databases in multi-omics technologies. Finally, we discuss the challenges of multi-omics analyses and their future prospects for drug-target identification, with a focus on the integration of transcriptome and proteomics.

## 2. Development History of Omics Technologies

**Figure 1:**
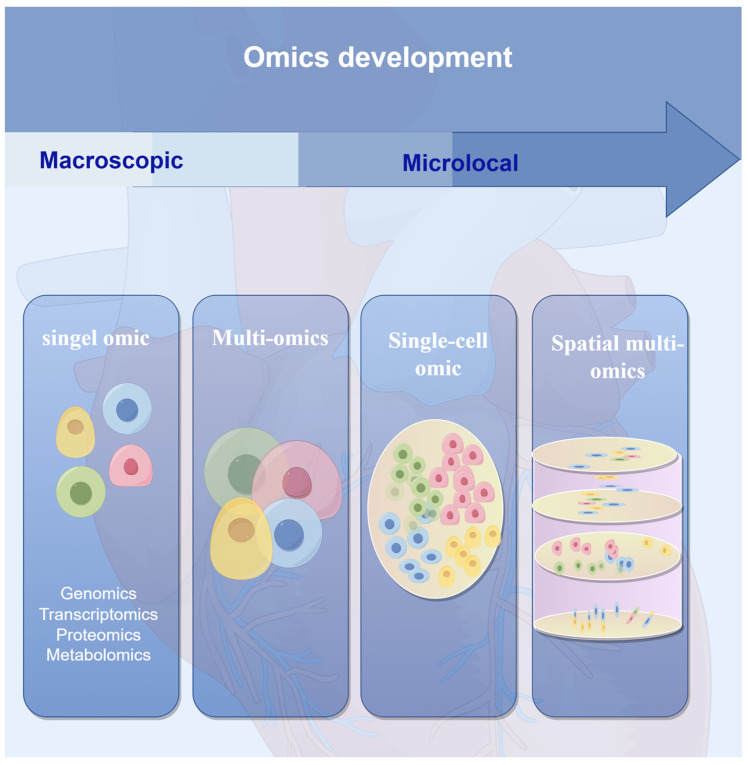
History of the development of omics technology.

Over the recent decades, continuous technological innovations have led to the emergence of various omics technologies, each presenting different but complementary aspects of bioinformatics. Omics data, including genomics, epigenomics, transcriptomics, proteomics, and metabolomics, have been used to identify and obtain vast amounts of single-dimensional data (Figure 1). This data helps dissect the molecular mechanisms of gene regulation or reveal various aspects of human diseases. Although the objectives of various omics studies differ, they are closely interconnected. According to the central dogma, DNA (genomics) transcribes into mRNA (transcriptomics), which is then translated into proteins (proteomics). These proteins can catalyze the production of or act on metabolites (metabolomics) and lipids (lipidomics) [10]. However, single-dimensional omics studies or data analyses alone cannot sufficiently explain how different multi-layered biological processes interact and give rise to complex phenotypes [11,12]. The results obtained may be limited by uncertainties related to specificity, selectivity, or biochemical or physiological relevance. Therefore, it is necessary to comprehensively capture and analyze multiple cellular processes to better understand the relationship between biological mechanisms and genotypic–phenotypic correlations.

With the application of high-throughput omics methods for the analysis of biological samples, there is a shift from single-omics-level research to comprehensive studies, transitioning from partial to holistic analyses. Consequently, the emergence of multi-omics integrative analysis is inevitable. Multi-omics is a novel approach and technology for systematically studying biology, integrating and analyzing data from multiple omics levels such as genomics, transcriptomics, proteomics, and metabolomics in an unbiased manner. This systematic approach dissects the mechanisms and phenotypes of complex biological systems [13,14]. Multi-omics integrative data analysis is not merely about stitching data together; it involves an in-depth exploration of biological explanations. By conducting comprehensive studies across multiple levels, including genetics, transcription, proteins, and metabolism, potential relationships and interactions can be discovered from data at different omics levels. Moreover, these findings can be mutually validated to reduce the limitations and false positives of single-omics analyses, thereby obtaining a more comprehensive biological explanation. This approach surpasses the information provided by single-omics analysis, enabling a more systematic study of the functionality and regulatory mechanisms of biological systems. It aids in constructing organismal regulatory networks and provides a deeper understanding of the interactions, regulations, and causal relationships among different levels. Additionally, it identifies key molecules and pathways in biological systems and discovers new biomarkers and therapeutic targets.

Multi-omics integrative analysis provides more comprehensive biological information than single types of omics data. These analyses are performed at the multicellular level, averaging cell signals and potentially overlooking minor differences, such as noise. In highly heterogeneous tissue samples, such as tumor tissues and immune cells, cell heterogeneity information may be lost, complicating the analysis of tumor characteristics from a cellular perspective. With the development of third-generation sequencing technology, single-cell sequencing technology has emerged to better understand cell heterogeneity and functional differences. In recent decades, advancements in high-throughput technologies and multi-omics integrative analysis have driven the emergence and development of single-cell multi-omics technology. Compared to single-cell genomics analysis, single-cell multi-omics analysis provides more insights into cell-type-specific gene regulation. The continuous development of single-cell technology has led to methods that enable the spatial localization of gene expression within tissues, introducing the concept of “spatial multi-omics”. Single-cell multi-omics analysis allows researchers to obtain transcriptomic, epigenomic, and proteomic information from individual cells. However, this process also dissociates samples from their native environments, disrupting the tissue structure and losing crucial information. To address these issues and identify cell types and distributions within complex tumor microenvironments, it is further necessary to determine the spatial positions and localizations of cells within tissues. Spatial transcriptomics technology was first proposed in 2016 and aims to address this challenge. Subsequently, spatial proteomics and other omics technologies have emerged, leading to the development of spatial multi-omics technology [15].

## 3. Single-Omics Analysis

### 3.1. Genomics

Genomics was initially proposed by American geneticists in 1986 to explore the composition, structure, function, localization, and editing of the genetic material DNA. The aim is to quantitatively study and analyze all genes within organisms for their biological significance. With the development of next-generation sequencing, genomic technologies can efficiently analyze whole-genome data to discover genes, proteins, and biological pathways related to diseases. For drug-target screening using genomic technologies, DNA-sequencing data from tumor and non-malignant tissue samples are compared to identify differential information distinct from that of the normal organism. These differential genes may lead to the identification of drug targets. Additionally, these genes can be combined with the CRISPR-Cas9 knockout technology to quantitatively screen the identified target genes individually [16,17].

Genomic research currently involves three main areas: structural genomics, functional genomics, and comparative genomics. Structural genomics focuses on analyzing nucleotide sequences using whole-genome sequencing techniques to determine the composition of the genome and the specific positions of genes. Functional genomics entails artificially altering the sequence or expression states of genes within the genome or cells and observing the resulting phenotype to establish the associations between genotype and phenotype, thereby clarifying the functions of genes. Comparative genomics investigates sequence variations by comparing the differences in genome structure and function among different species and their inherent connections [18]. Functional genomics, through the study of gene functions and gene networks, has emerged as a key tool for deciphering the complex composition and diverse effects of human tumors and their microenvironments. Technologies in functional genomics, such as RNA interference [19,20], small interfering RNA [21], short hairpin RNA [22], CRISPR interference, and CRISPR inhibition [22,23], play important roles in drug-target discovery and validation. Despite offering new perspectives for disease diagnosis and treatment, genomics has limitations in predicting changes in protein and metabolic levels in organisms. Increasingly, research reveals that DNA and RNA alone cannot fully illuminate the function and status of proteins [24]. The correlation between protein and mRNA expression levels in mammals is relatively low, approximately 0.40. Additionally, due to the high heterogeneity of tumor cells, the failure of targeted drugs is also a challenge. For instance, mutations identified by gene-sequencing technology are often not “driver mutations”, which leads to unsatisfactory results of targeted drugs. Currently, the number of people benefiting from gene-based targeted therapeutic drugs remains relatively small [25].

### 3.2. Transcriptomics

The concept of the transcriptome was initially introduced by Charles Auffray in 1999 [20]. Transcriptomics refers to the study of gene transcription and transcriptional regulation at the overall cellular level, specifically exploring the dynamic changes in gene expression from DNA to RNA. Compared to genomic research, transcriptomics technologies exhibit greater complexity and diversity, revealing spatiotemporal differences in gene expression. By analyzing transcriptomic data from tumor and non-malignant tissue samples using high-throughput sequencing technologies, such as RNA sequencing, researchers can uncover distinct gene-expression patterns in tumor cells. This enables the identification of genes significantly upregulated or downregulated in tumor tissues, thereby determining potential drug targets. For instance, in oncology, comparing the transcriptomes of tumor cells and normal cells allows researchers to identify genes specifically overexpressed in cancer, which are often closely related to cancer growth and metastasis and can serve as candidate targets for targeted therapy [26]. Additionally, transcriptomic analysis can be utilized to monitor the effectiveness of drug treatments by analyzing the changes in gene expression before and after treatment, thereby evaluating the mechanism of action and the efficacy of the drugs [27]. Currently, available transcriptomic sequencing technologies include mRNA sequencing [28]. Other transcriptomic sequencing technologies encompass long non-coding RNA (LncRNA) sequencing, circular RNA sequencing, whole transcriptome sequencing, and single-cell transcriptome sequencing [29,30,31].

### 3.3. Proteomics

Proteomics was initially introduced in 1994 by American scientist Marc Wilkins [32]. Proteomics is the scientific study of the protein complement of a cell, tissue, or organism, encompassing its composition and variations over time [33,34]. Unlike the genome, the proteome can vary with the tissue and even environmental conditions. The process of transcribing genes into proteins involves a series of complex post-transcriptional regulatory mechanisms, such as mRNA splicing and post-translational modifications (PTMs), which contribute to the increasing complexity and diversity of proteins [35,36]. The majority of known drug targets are proteins, and using proteomics data to identify candidate drug targets can significantly increase the likelihood of drug approval compared to genomics [37]. Hence, identifying drug targets at the protein level is a crucial direction in drug development. The common strategy for target discovery in proteomics is based on target-based discovery for drug development [38,39]. By comparing the expression differences of proteins between the diseased and normal physiological states, potential drug targets can be identified. This principle relies on high-throughput protein detection and quantification technologies, primarily mass spectrometry (MS) and protein affinity purification techniques. Among them, proteomic analysis based on high-throughput MS is commonly used for target screening. By separating, identifying, and quantifying protein samples, differences in protein expression under physiological and pathological conditions can be revealed, identifying differential proteins as potential drug targets. Another proteomic technique is protein affinity purification, such as immunoprecipitation and affinity chromatography, which accurately identifies proteins relevant to drug action by specifically interacting with other biomolecules. The strategy of target discovery through chemical modification of small molecule probes includes two main methods: compound-centric chemical proteomics [40] and activity-based protein profiling [41].The label-free affinity chromatography methods include cellular thermal shift assay [42] and thermal proteome profiling [43], drug affinity responsive target stability [44], limited proteolysis mass spectrometry, and stability of proteins from rates of oxidation [45]. Researchers can screen differential proteins from cells using MS techniques and then validate the protein components in these complexes through protein affinity purification, discovering potential drug targets. For instance, in the proteomic analysis of cerebrospinal fluid EVs from four Alzheimer’s disease (AD) patients and normal controls, 11 significantly differentially expressed EV proteins were identified among 1765 proteins. Subsequently, an enzyme-linked immunosorbent assay was then used to validate a significantly different indicator, Cat B, in 136 samples. This led to the discovery that EV-CatB may serve as a candidate biomarker for the pathological staging of AD [46].

In recent years, the application of modified proteomics in life sciences has significantly increased in addition to traditional proteomics [47]. PTMs of proteins involve covalent alterations of amino acid residues to add modification groups or through alternative splicing [48,49]. PTMs play crucial roles in regulating cellular physiological functions, including phosphorylation, acetylation, and glycosylation [50]. Although proteomics technology offers a robust platform for investigating protein complexity within organisms, it remains in an early stage of development. However, advancements in high-throughput MS are gradually unveiling the vast potential of proteomics in life sciences and drug development.

### 3.4. Metabolomics

Metabolomics refers to the qualitative and quantitative measurement of dynamic changes in metabolites within living systems caused by pathological, physiological, or genetic alterations [51]. Unlike studies that solely focus on gene expression or proteomics, which reveal only a portion of cellular behavior, metabolomics depicts the complete physiological state of a biological component or cell at a specific moment. Thus, metabolomics offers direct evidence for understanding physiological and pathological processes, as well as the molecular mechanisms of disease metabolism. Compared to proteomics, metabolomics analysis has the advantages of simplicity and rapidity. Metabolomics involves the qualitative and quantitative analysis of all low molecular weight metabolites (<1500 Da, such as amino acids, sugars, and lipids) within cells or organisms using liquid chromatography–mass spectrometry (LC-MS), gas chromatography–mass spectrometry, or nuclear magnetic resonance (NMR). This approach aims to uncover the types and changes of metabolites and identify their relative relationships with physiological and pathological alterations [52]. Based on research objectives, metabolomics can be divided into untargeted and targeted metabolomics [30].

Untargeted metabolomics is the most commonly used approach in metabolomics applications, aiming to comprehensively detect the entire metabolome of an organism to identify potential biomarkers [53]. It focuses on identifying significant metabolic features that differ between the experimental and control groups and then interprets the discovered metabolites and their metabolic pathways in relation to biological processes. Conversely, targeted metabolomics focuses on specific metabolites as research targets, which can be used for validating biomarkers. Currently, there are four common strategies for metabolite discovery in metabolomics: 1. MS based: utilizing MS to detect and identify specific metabolic changes in diseased states; 2. sequencing techniques based: Although primarily used for genetic information, some techniques can assist in deciphering metabolic pathways; 3. bioinformatics approaches based: by conducting extensive bioinformatics analyses to identify patterns or rules related to specific diseases from large sets of metabolomics data, thereby discovering disease targets; and 4. animal model experiments based: by simulating human disease symptoms in animal models, it is possible to observe which metabolites or metabolic pathways change, and by analyzing these differential signals, disease targets can be identified [54].

## 4. Classic Multi-omics Techniques Analysis

Biological processes are characterized by their complexity and integrality. The analysis of single-omics data often fails to clarify complex causal relationships. However, integrated multi-omics analysis can simultaneously explore biological questions from both causal and effect perspectives and verify their correlations. Correlation analysis is a practical technique for uncovering associations within large datasets, thereby depicting patterns and trends where certain attributes of a phenomenon change simultaneously. Multi-omics correlation analysis typically begins by addressing intra-group differences (phenotypic differences), including spatiotemporal variations in sample collection, differences in disease or health status among samples, and the presence or absence of sample processing operations. Based on inter-group differences, inter-group correlation analysis detects associations or correlations within large datasets. This integration reveals interactions and regulatory relationships among data from different levels, facilitating comprehensive multi-level investigations of diseases [10,55,56].

**Figure 2:**
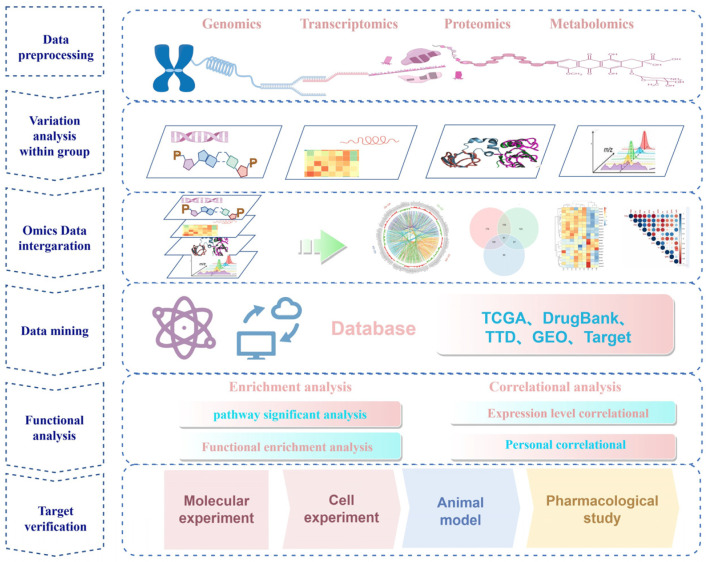
Multi-omics technology is applied to drug-target identification process.

**Figure 3:**
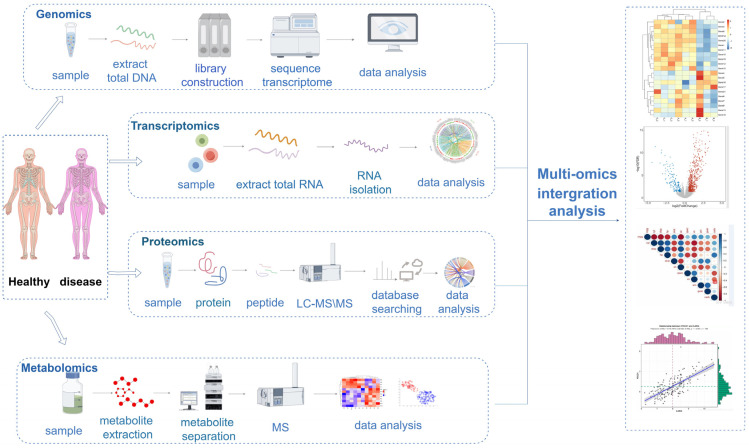
Single-omics analysis steps and multi-omics analysis.

Multi-omics analysis methods (Figure 2) generally encompass correlation analysis and enrichment analysis. The integration approach for correlations involves directly analyzing the correlation between two or more omics datasets. Initially, differential information is obtained through single-omics sequencing, and then, various omics data are connected for correlation analysis (Figure 3). For instance, overlap analysis can be conducted using Venn diagrams, which count the common or unique differentially expressed genes/proteins in multiple omics datasets, providing an intuitive understanding of the similarity and overlap between groups. Pearson correlation analysis can be employed to measure the relationship between inter-group variables and the extent of this relationship. When the data do not follow a normal distribution, Spearman correlation analysis is typically used. Visualization can be achieved using heatmaps and scatter plots. When the correlation between two omics datasets is weak, a nine-quadrant plot analysis can visually display the correlation, assisting researchers in identifying key molecules relevant to the study. For example, through a nine-quadrant plot analysis of the transcriptome and proteome, researchers can comprehend the expression of genes in samples and the translation of gene expression, facilitating the identification of the key genes relevant to the study. In one study, the authors used tandem mass tag proteomics technology to analyze protein changes in hepatocellular carcinoma cells after knocking down small Cajal body-specific RNA 13 (*SCARNA13*) and discovered 182 differentially expressed proteins. An overlap analysis using Venn diagrams revealed 11 genes that were differentially expressed in both transcriptomics and proteomics, with the protein-expression level of SRY-related HMG-box gene 9 significantly downregulated after *SCARNA13* knockdown [57].

Another multi-omics analysis method is enrichment analysis. Biological processes entail the coordinated participation of multiple genes/proteins. Hence, abnormalities in a biological process are the consequence of interactions among multiple genes. Molecular responses and alterations within organisms demonstrate functional and pathway enrichment. Accordingly, different omics datasets also exhibit similar patterns of functional enrichment and changes. Differential proteins/genes from diverse omics datasets can generate a considerable amount of data through direct annotation. These functions often overlap conceptually, resulting in redundant analysis outcomes and impeding further refined analysis. Therefore, using enrichment-analysis methods to filter and screen data, integrating data with overlapping functions, and identifying significantly overexpressed genes/proteins or differential genes/proteins compared to background sets in biological composition/functions can generate more meaningful functional information. For instance, the authors enriched significantly upregulated genes in each subtype and discovered that Nudix hydrolase 12, Acetyl-CoA synthetase 1, and Nicotinamide nucleotide Adenylyl transferase 3 in the S3 subtype are all related to nucleotide biosynthesis processes [58]. Currently, there are two most commonly used enrichment-analysis methods. One is to set a significance threshold, select differentially expressed genes, and then utilize statistical tests to determine whether these differential genes are enriched in specific functional categories or pathways. Herein, pathway enrichment is typically selected for gene ontology (GO), the Kyoto Encyclopedia of Genes and Genomes (KEGG) database, or other defined pathway enrichment. This is known as GO enrichment analysis or KEGG enrichment analysis. However, subjective threshold setting may neglect genes with significant biological importance that are not significantly differentially expressed. The emergence of gene set enrichment analysis (GSEA) addresses this deficiency. GSEA does not require the specification of a specific threshold for differential genes. By concentrating on the coordinated pattern of changes in the entire gene set, even genes with minor expression changes can be effectively captured by GSEA as long as they produce a synergistic effect within the entire gene set. The annotation information therein can be derived from GO, KEGG, or any other format-compliant information.

## 5. Classical Multi-omics Integration Methods

The integration of multi-omics data is of crucial significance for target screening and identification. Different impacts are generated when conducting inter-omics integration analysis with various omics. The integration of genomics and transcriptomics is capable of revealing the association between gene mutations and gene expression, facilitating the study of transcriptional regulation and enabling the identification of potential drug targets. The integration of transcriptomics and proteomics can expose the correlation between gene expression and protein levels, thereby uncovering gene expression at two levels. The integration of proteomics or transcriptomics with metabolomics can delve into the relationship between protein expression and metabolite levels and detect the association between phenotype and genotype, thereby identifying the upstream regulatory mechanisms of metabolic characteristics via a “from effect to cause” approach. The integration of proteomics and proteomics modification analysis can explore the true mechanism of protein modification from two dimensions, namely protein expression and modification (Figure 3).

### 5.1. Integrating Transcriptome and Proteomics

As downstream products of genetic and epigenetic regulation, the transcriptome and proteome respectively measure gene expression at the transcriptional and translational levels, indicating a very close connection. The transcriptome acts as an intermediate module connecting the genome and the proteome, is capable of identifying genes with differential expression after being treated with small molecules, and is used to formulate hypotheses about mechanisms such as transcriptional and post-transcriptional regulation [25]. The process of gene transcription and translation into proteins involves complex post-transcriptional regulatory mechanisms, such as mRNA splicing and PTMs of proteins [35]. Therefore, insights into proteins cannot be sufficiently obtained from the DNA and RNA levels alone. Proteomic and transcriptomic analyses provide insights regarding the regulation of protein abundance. Proteins, as the direct executors of biological functions, reveal correlations that overlap with but are not identical to transcriptomic and genetic data, representing a true reflection of gene-expression status. The development of high-throughput MS technology has enabled the large-scale study of the characteristic information of all proteins expressed in a cell or organism.

Integrating transcriptomic and proteomic analyses enables the assessment of gene expression both upstream and downstream, as well as the exploration of gene regulation during transcription and translation processes. By comparing samples from diseased and normal physiological states, genes and proteins with significantly altered expression can be identified. Exploiting the differences and complementarities between the transcriptome and the proteome allows for expression regulation and correlation. Through GO enrichment analysis and KEGG functional enrichment analyses, researchers can gain insights into the specific functions of these molecules in diseases and their interaction patterns. These functional enrichment analyses help uncover key molecular pathways that affect specific biological functions, particularly those that may involve new drug targets or therapeutic mechanisms. The integration of transcriptomic and proteomic analyses enables the construction of a protein search library from transcriptomic data, enhancing the completeness of the protein database and significantly improving the accuracy of protein identification. Conversely, proteomics can validate alternative splicing information discovered through transcriptomics. By comparing the protein-expression differences between diseased and normal physiological states, potential drug targets can be identified. For instance, in the research on therapeutic targets for non-alcoholic fatty liver disease (NAFLD), researchers used comprehensive proteomic analysis to investigate the differential expression profiles of ER stress-response proteins under various metabolic states. They discovered a significant downregulation of major urinary protein (MUP). Integrated transcriptomics further confirmed this finding at the mRNA and protein-expression levels, revealing that MUP1, as the primary secretory form of MUP, decreased under endoplasmic reticulum stress in hepatocytes. This indicates the potential of recombinant MUP1 or its derivatives produced under endoplasmic reticulum stress as promising therapeutic targets for alleviating NAFLD [59]. Additionally, by conducting transcriptomic, proteomic, and phospho-proteomic analyses on a silicosis mouse model, researchers constructed a multi-omics integrated analysis map, thereby tracking the dynamic changes caused by silica-induced injuries. These analyses confirmed the presence of abnormal pathways in the progression of silicosis, including transcriptional, proteomic, and kinase activities, particularly identifying the phosphorylation levels of epidermal growth factor receptor and spleen tyrosine kinase as new therapeutic targets for silicosis [60]. Furthermore, in analyzing the expression levels of *integrin subunit alpha 2* (*ITGA2*) in *Kirsten rat sarcoma viral oncogene* (*KRAS*)-mutant pancreatic cancer, researchers observed a significant increase. Subsequent reverse transcription-polymerase chain reaction and Western blot analyses confirmed that aberrant *KRAS* activation induced overexpression of *ITGA2*. Single-cell RNA sequencing (scRNA-seq) data revealed that *ITGA2* expression regulates the activation of the small mothers against decapentaplegic 2-inhibited transforming growth factor-β (TGF-β) signaling pathway, potentially serving as a clinical therapeutic target by enhancing the anti-tumor effects of TGF-β [61].

### 5.2. Integrating Transcriptome and Metabolomics

Transcriptomics and metabolomics, being the upstream and downstream products in omics research, respectively, measure gene expression at both the transcriptional and metabolic levels of the same gene. Transcriptomic investigations focus on the transcriptional state of specific genes in a particular tissue or cell, along with the transcriptional regulatory patterns. Metabolomic studies aim to comprehend the metabolic networks of organisms and explore the roles of metabolites in the occurrence and treatment procedures of diseases. As direct manifestations of phenotypes, even minor changes in phenotypic traits can be exponentially magnified at the metabolic level. Hence, alterations in metabolites can reflect changes in phenotype and explain the mechanisms influencing the relationship between phenotype and metabolites. However, downstream metabolomics can only detect the composition of metabolites in cells and metabolic tissues. Single-omics techniques alone struggle to identify the specific upstream molecules that regulate changes in metabolomics. Therefore, by integrating metabolomics with transcriptomic analysis, we can observe how changes in phenotype are manifested at the gene level. Additionally, association analysis of phenotypic and genotypic data allows us to infer the upstream “causes” from the downstream “results”, thereby discovering the regulatory mechanisms of characteristic metabolites upstream.

By fully leveraging the correlation between metabolomic data and transcriptomic and genetic data, which overlap but are not identical, a comparative analysis of the two omics data can reveal molecular information at both omics levels. Starting with the analysis of differential metabolites and genes from metabolomics and transcriptomics, respectively, single-omics analysis can be performed. Methods such as principal component analysis, orthogonal projections to latent structures discriminant analysis, and clustering analysis can achieve intra-group differentiation, and differential molecules can be identified through differential comparison analysis. After identifying differential molecules, metabolomics can confirm the associations between differential metabolites and pathways via KEGG pathway enrichment analysis. Transcriptomics can perform KEGG enrichment analysis, GO enrichment analysis, and protein–protein interaction networks analysis. By integrating the KEGG data from these two omics, relevant functions or shared pathways can be identified and key pathways found. Alternatively, by targeting metabolites associated with upstream pathways identified by transcriptomics, specific mechanisms of upstream regulation can be investigated through metabolite detection. Expression correlation analysis can also be conducted between the differential genes and the accumulated differential metabolite information obtained from transcriptomic and metabolomic analyses to identify their expression correlations and infer their involvement in the same biological processes. Differential metabolites and metabolic pathways can be identified, and correlation analysis of these differentially expressed metabolites can be conducted to identify related biomarkers. This approach allows for the exploration of biological questions from both the “cause” and “effect” perspectives, facilitating the diagnosis of related diseases and hierarchical prediction, among other applications. Elke Schaeffeler et al. [62] utilized human leukocyte antigen ligandome analysis based on MS to identify clear-cell renal cell carcinoma (ccRCC)-associated peptides, followed by integrated transcriptomic and metabolomic analysis to further screen ccRCC targets. Differential gene enrichment analysis using gene set enrichment analysis on the entire transcriptome gene set led to 113 final candidate genes. The study employed LC-MS for the targeted metabolomics analysis of tissue samples, quantifying 204 tumor metabolites. Non-targeted metabolomics analysis was then used to identify metabolic products, revealing the selected therapeutic target prolyl hydroxylase 3 (PHD3; EGLN3). Similarly, Haitao Lu et al., adopted a precise targeted metabolomics approach to identify for the first time that the crucial functional metabolites adenosine monophosphate (AMP) and cyclic adenosine monophosphate (cAMP) significantly accumulated in gemcitabine-induced mouse pancreatic cancer (PC) tumor tissues. Subsequently, quantitative proteomic and transcriptomic analysis validated a large number of metabolic enzymes and genes associated with alterations in AMP and cAMP. Finally, it was verified that the accumulation of AMP and cAMP induced by gemcitabine can, respectively, activate the downstream kinases AMP-activated protein kinase and protein kinase A, leading to tumor growth inhibition through the overexpression of growth arrest and DNA damage-inducible protein 45A. The dual activation of the AMP-cAMP axis represents a potential new target for PC treatment [63].

### 5.3. Integrating Proteomics and Metabolomics

As downstream products of omics research, proteomics and metabolomics, respectively, represent the complete set of proteins and metabolites expressed by a cell or an entire organism. Proteomics studies the composition and alteration patterns of proteins within cell tissues or organisms. Metabolomics examines the small-molecule metabolites involved in regulation, including their types, quantities, and patterns of change. Metabolomic information is closely aligned with biological phenotypic characteristics. According to the central dogma, the expression of metabolites, following transcription and protein translation, is ultimately manifested in the phenotype. Metabolomics is downstream of proteomics and represents a further manifestation of proteins. Thus, as two directly related groups in regulatory relationships, minor functional alterations in the proteome may be significantly amplified several times at the metabolic level. Integrating proteomics with metabolomics allows these methods to validate and complement one another, clarifying how proteins govern specific changes in metabolites and illuminating their enhancements at the genetic level. This integration also provides insights into the impact on phenotypes and the establishment of subsequent molecular mechanisms.

Similar to an integrated analysis involving transcriptomics and metabolomics, combining proteomics and metabolomics in the analysis of biological samples enables KEGG integration analysis and expression correlation analysis based on the co-expression patterns of metabolites and proteins. In KEGG integration analysis, differential metabolite and protein data are rapidly screened to identify common involvement in metabolic pathways, assisting in elucidating how proteins regulate metabolites and their engagement in signaling pathways. Expression correlation analysis, based on the expression levels of selected differential metabolites and proteins, identifies differential proteins and metabolites with similar trends. This clarifies how changes in a protein can result in changes in metabolites and other associated changes. Additionally, orthogonal two partial least squares (O2PLS) modeling analysis, also known as orthogonal partial least squares discriminant analysis of paired data, is conducted. Unlike traditional one-to-one correlation analysis, O2PLS conducts one-to-many operations on the expression levels of the two omics datasets. It starts from the overall data perspective and then undertakes bidirectional modeling of the two omics datasets as a whole. It predicts datasets with potential correlations between the two matrices, thereby identifying correlations between a protein and metabolites.

Zongwei Cai and colleagues [64] carried out a systematic chemical proteomics study on the target proteins of perfluorinated compounds. Through quantitative proteomic analysis of the enriched proteins, acetyl-CoA carboxylase alpha and acetyl-CoA carboxylase beta were identified as new target proteins of perfluorooctanoic acid (PFOA). Subsequently, targeted proteomic studies using parallel reaction monitoring further verified these protein targets. In combination with metabolomics research, the verification of the in vivo metabolic changes selected by the differential target proteins provided a rational explanation for the liver toxicity induced by PFOA. Eventually, the true protein targets of PFOA were disclosed and validated. Hui Zhong et al. [65] utilized phosphorylation proteomics and proteomic analysis to explore the ability of the recombinant Golgi protein 73 (GP73) to promote guanosine triphosphate hydrolysis. They found that GP73 impairs the secretion of apolipoprotein B (ApoB) and ApoB100, while GP73 mutants nullify these effects. The metabolomic exploration of the differential protein data further evaluated the metabolic consequences of chronic GP73 expression in liver cells, clarifying the abnormal biological phenomena in pathological states. It was observed that mice with chronically upregulated GP73 in liver cells showed a phenotype characteristic of non-alcoholic fatty liver disease (NAFLD), and treatment with metformin to inhibit GP73 GTPase-activating protein activity effectively blocked the GP73-induced non-obese NAFLD phenotype. This finding suggests that elevated levels of GP73 may promote the progression from steatosis to nonalcoholic steatohepatitis, providing a potential therapeutic target for halting the development and progression of non-obese NAFLD.

### 5.4. Proteogenomics

The term “proteogenomics” was initially proposed by Jaffe et al. in 2004 [66]. It encompasses the integration of MS-based proteomics and PTM proteomics with genomic, epigenomic, and transcriptomic data. Genomics and epigenomics mainly involve the collective characterization and quantification across the entire genome, providing a blueprint of cellular events. By studying specific genes, we can uncover gene variations related to diseases [67]. This comprehensive approach reveals the pathogenic mechanisms of human diseases, identifies differential genes, and discovers potential targets for diagnosis and treatment. According to the central dogma, after DNA transcription and protein translation, the expression of metabolites ultimately presents phenotypically; the implementation of biological functions in organisms ultimately depends on proteins and their metabolites. Proteins, as the direct executors of biological functions, provide confirmation of events that have already occurred, as proteins and their modifications are the main determinants of biological phenotypes. Their composition and interactions form the foundation of the dynamic processes of life. Therefore, monitoring proteins and their interactions is crucial for studying life activities [68]. Compared to genomics, proteomics has the advantage of confirming events that have already happened. Thus, genomic and transcriptomic analyses provide characteristics of differential genomic features, while proteomics can directly identify protein regulation and signal transduction responses to differential genes. Additionally, an in-depth quantitative analysis of post-translationally modified proteins is carried out. The integrative analysis of multi-omics data, such as genomics, transcriptomics, and proteomics, enables the exploration of biological questions from both causal and consequential perspectives. Genomic and transcriptomic analyses provide characteristics of differential genomic features, while proteomics can directly identify proteins regulating and responding to abnormal expressions and provide direct information on signal transduction. Using MS for in-depth quantitative analysis of post-translationally modified proteins can detect genomic changes and signal dysregulations that genomics and transcriptomics cannot reveal [69,70]. The proteogenomics analysis workflow starts with transcriptome sequencing to obtain gene information, coding transcripts, LncRNA, alternative splicing sites, single nucleotide variants (SNVs), etc. from the genome reference sequence to construct a custom feature column database or utilize public databases for assistance. Then, protein-expression data is obtained through MS-based proteomic analysis. The spectrum identification engine matches the collected MS data with peptides in the previously constructed database to score peptide-spectrum matches. Unlike the traditional protein identification quality-control process, the new feature sequence database contains a large amount of redundant and random sequences. Therefore, more stringent quality-control standards and validation methods are needed to ensure that the identified new sequences are sufficiently reliable. The identified new peptides are screened and classified, and the number of different events is counted. A manual inspection is conducted to assist in verifying the reliability of each event. Finally, the genomic localization of events is completed, and data visualization is performed.

Researchers from the Clinical Proteomic Tumor Analysis Consortium analyzed the proteogenomics of lung adenocarcinoma (LUAD) through a multi-omics approach and established different subtypes of LUAD based on their distinct immune characteristics. Phospho-proteomic analysis identified anaplastic lymphoma kinase fusion as a potential diagnostic biomarker and target [71]. Additionally, the research group led by Yu-Ju Chen [72] conducted proteogenomic analysis on non-smoking LUAD patients in East Asian populations. They identified the age, gender, and environmental carcinogenic risk factors associated with the development of LUAD. Moreover, they clinically classified early-stage LUAD based on proteomic features and identified tumor characteristics, tumor cell markers, and drug targets via protein network analysis. Tan Minjia and colleagues [73] conducted an in-depth analysis of the proteomic expression profiles and phosphor-proteomic profiles of LUAD and the adjacent tissues from 103 clinical patients. They identified a total of 11,119 protein products and 22,564 phosphorylation modification sites. Furthermore, by integrating a genomic feature data analysis, they confirmed that heat shock protein 90β could serve as a prognostic biomarker for LUAD by using proteomic features [74].

## 6. Single-Cell Multi-omics Technology

Integrative analysis of multi-omics offers more comprehensive biological information compared to single-type omics data. These omics sequencing techniques are conducted at the multicellular level, resulting in averaged signals of cells, which may neglect minor differences as being noise. For highly heterogeneous tissue samples, such as tumor tissues and immune cells, the information regarding cell heterogeneity is lost, making it difficult to analyze tumor characteristics at the cellular level. To better understand the heterogeneity and functional differences of cells, single-cell sequencing technology has emerged. Single-cell multimodal omics technology refers to the cutting-edge technique of depicting cell heterogeneity through multi-dimensional, multi-level, and multi-angle genomic, epigenomic, transcriptomic, and proteomic analyses within the same cell, enabling the exploration of direct and potential connections among various omics layers and facilitating a more accurate and comprehensive explanation of disease states [75,76,77,78].

Conventional single-cell sequencing involves several key steps: preparing single-cell suspensions, single-cell sorting, library preparation, high-throughput sequencing, bioinformatics analysis, and data visualization. First, a single-cell suspension is prepared by dissociating tissue samples into individual cells, which allows for the extraction of nucleic acid information from each cell. The nucleic acids (RNA, DNA, or proteins) within each cell are then captured. This is followed by the amplification of the obtained DNA or RNA, library construction, and finally, data analysis. Each step—from isolating, fixing, and lysing single cells to amplifying nucleic acids and sequencing—is meticulously designed to preserve the integrity and accuracy of the molecular information from each cell [15,38,77].

Currently, the most established methods in single-cell omics are single-cell transcriptomics and single-cell genomics [79]. Recent progress has been made in single-cell sequencing technologies. For instance, single-cell genomics utilizes whole-genome sequencing to analyze the entire genomic DNA sequence of individual cells, identifying gene mutations, chromosomal structural variations, and copy number variations. Single-cell transcriptome sequencing analyzes the mRNA expression in individual cells, revealing the differences in gene expression among cells and identifying different cell types and functional states. Popular scRNA-seq technologies include Smart-seq2, which provides full-length transcript information, and 10X Genomics Chromium, which can handle thousands of single cells with high throughput. Single-cell epigenetic sequencing analyzes DNA methylation and histone modifications in individual cells to understand the epigenetic mechanisms of gene regulation. This involves single-cell whole-genome bisulfite sequencing for analyzing DNA methylation patterns and single-cell chromatin accessibility sequencing (such as single-cell assay for transposase-accessible chromatin using sequencing; scATAC-seq) for assessing chromatin openness and inferring regulatory element activity. Integrating single-cell omics technologies allows for a comprehensive understanding of cell states and functions, providing an unbiased exploration of the relationship between gene expression and phenotypic heterogeneity [80].

Single-cell multi-omics sequencing combines data from various single-cell omics, such as genomics, transcriptomics, and epigenomics, to provide a comprehensive view of cell states and functions. This approach involves sequencing the same cell using multiple single-cell techniques and then integrating the data through fusion and comprehensive analysis to uncover the diverse biological properties of cells. By merging information from multiple omics layers, including DNA, RNA, and protein data, researchers can improve the accuracy of identifying cell populations, tracing cell lineages, and detecting new or rare cell types [78]. Several methods have been developed for single-cell multi-omics analysis of the genome and transcriptome. For instance, the single-cell combinatorial indexing for methylation analysis technique can simultaneously capture transcriptomic and epigenomic information from a single cell. Conducting a parallel analysis of the genome and a transcriptome in the same cell reveals the transcriptional state of the genome, highlighting the relationship between genomic changes and the transcriptional outcomes of the target genes involved in disease processes. Integrating transcriptomic and epigenomic data can directly clarify the epigenetic features of DNA. Additionally, combining transcriptomic and proteomic analysis at the single-cell level allows for the characterization of proteins through PTMs and interactions, improving the analysis of mRNA and protein abundance relationships and enhancing identification accuracy. For example, researchers [81] combined scRNA-seq, single-cell chromatin accessibility analysis, and DNA sequencing to analyze 225 tumor samples from 11 different types of cancer. They created a multi-omics tumor atlas and identified the genetic factors associated with cancer. Notably, they found that transcription factors could serve as markers for cancer prognosis. Researchers [82] conducted detailed transcriptomic and epigenetic analyses on 42 and 39 human cancer cell lines, respectively, using scRNA-seq and ATAC sequencing technologies. This study uncovered significant heterogeneity at both the transcriptomic and epigenetic levels. Additionally, integrating data from multiple omics layers, including DNA, RNA, and proteins, enhances the accuracy of identifying cell populations, tracing cell lineages, and detecting new or rare cell types. However, preparing single-cell suspensions and isolating individual cells remains a challenging aspect of single-cell omics technologies.

Although the throughput, automation, and detection speed of single-cell sequencing technologies have been significantly enhanced, and the costs have been continuously decreasing, they still fail to meet the requirements of clinical testing. The application of single-cell multi-omics analysis is still in its early stages. While single-cell transcriptomics is more developed, other types of single-cell multi-omics analyses are still evolving, and there is a need to improve the methods for analyzing single-cell multi-omics data. For instance, as proteins cannot be amplified like DNA or RNA, and the current instruments lack sufficient sensitivity, the number of proteins detectable with the current single-cell multi-omics technologies is limited, which slows down the development of single-cell proteomics. Currently, there are few cases of using single-cell multi-omics alone for drug target screening; it is usually combined with other omics technologies. For example, traditional omics can be combined with single-cell omics to validate potential targets. Initially, potential targets are identified based on extensive omics data, and then, single-cell omics data is used to further refine these targets. Alternatively, spatial multi-omics technology can be integrated with single-cell omics techniques [41].

Furthermore, there are few computational methods available for the integrated analysis of single-cell multi-omics data, and some technical and computational limitations remain. Although the recent progress in scRNA-seq technology has led to an exponential increase in the number of cells and genes detected, suitable data-analysis methods for other types of single-cell omics, such as single-cell proteomics and single-cell metabolomics, are still lacking and need improvement. Single-cell omics sequencing technologies are not yet ideal for studying human cells. This is particularly true in stem-cell research, where it is currently impossible to identify the spatiotemporal changes of stem cells within the human body. In the future, combining single-cell multi-omics sequencing with gene editing tools and 3D organoid culture systems may potentially uncover the genetic changes in stem cells and their potential links to phenotypic changes in stem cells [79].

## 7. Spatial Multi-omics Technology

As single-cell omics become more prevalent, the single-cell dimensional atlas alone can no longer satisfy the diverse demands for in-depth research on disease treatment targets. Single-cell multi-omics analysis enables researchers to extract information regarding transcriptomics, epigenomics, and proteomics from individual cells. However, this approach isolates samples from their native environments, disrupting tissue structure and losing crucial information [15]. To address these challenges and accurately identify cell types and distributions in complex tumor microenvironments, it is crucial to determine the spatial positioning of cells within tissues. New methods that can spatially map gene expression within tissues have propelled the concept of “spatial omics.” The technology of spatial transcriptomics was first introduced in 2016. Since then, other technologies, such as spatial genomics and spatial proteomics, have emerged, giving rise to the development of spatial multi-omics techniques. During this time, spatial transcriptomics has also seen rapid advancements. In 2020, *Nature Methods* recognized this technology as the Method of the Year [83]. In 2022, this technology was listed as one of the top seven technologies by *Nature* [84]. For example, Michael T. Longaker [85] utilized single-cell transcriptome sequencing and single-cell chromatin accessibility data, along with 10x Visium spatial transcriptomics and PhenoCycler-Fusion spatial proteomics, to study various solid tumor types across different species. This comprehensive approach revealed new therapeutic targets specific to cancer-associated fibroblasts, regardless of species and tumor type. Sun [86] used a combination of spatial metabolomics and spatial transcriptomics to analyze slices of gastric cancer tissue. This method mapped how metabolites, lipids, and genes interact and co-locate within the metabolic pathways of diverse cancerous tissues. Currently, since spatial multi-omics technologies lack single-cell resolution, they typically only analyze clusters of about 3–10 cells. As a result, these technologies are often paired with single-cell holography techniques for more detailed insights. Abhay Kanodia [87] conducted a transcriptomic analysis of colorectal tumors, combining spatial transcriptomics at the tissue level using DSP technology with scRNA-seq. They cross-validated the results of several key processes related to immunotherapy across various cell types, confirming the coordinated changes in key processes of multicellular interactions within the tumors.

Spatial multi-omics technology combines biological data from various levels, such as DNA, RNA, and proteins, through a systems biology approach. It emphasizes the spatial characteristics and resolution of omics data, facilitating a comprehensive understanding of biological processes in their spatial context. This technology offers researchers a new three-dimensional spatial perspective, enhancing the “resolution” of cellular and genetic maps and addressing spatial heterogeneity within samples. In the next five to ten years, spatial multi-omics is anticipated to be widely adopted, potentially revolutionizing our understanding of the complexity of life by uncovering the intricate molecular and spatial structures of tissues and cells. Spatial metabolomics utilizes MS imaging combined with metabolomics, providing both qualitative and quantitative analysis of metabolites while revealing molecular phenotypes in a spatial context. Spatial proteomics technology employs high-precision laser-capture microdissection to isolate specific tissue regions or cells of interest. Using optimized micro proteomics techniques, it analyzes protein expressions at different spatial locations, obtaining protein-expression profiles for various functional areas and cell types within the sample. This helps discover more precise biomarkers and functional mechanisms. Spatial transcriptomics combines the detailed morphological observations of histology with the high-throughput sequencing capabilities of transcriptomics, enabling the simultaneous high-throughput sequencing of multiple sites on a single tissue slice.

The utilization of spatial omics technology has been steadily increasing, mapping the genomic architecture across various tissues. However, we are still in the early stages of the spatial omics era. As these methods progress, the limitations, in terms of throughput, resolution, sensitivity, and adaptability to various sample types, are gradually being overcome. Substantial progress in spatial omics is anticipated in the future, which will further deepen our understanding of the complexity of life.

At present, data acquisition is still one of the most challenging aspects of spatial omics methods. The vast data volume complicates spatial analysis and requires significant effort from researchers for data interpretation. Currently, spatial transcriptomics technology operates on a “2D” level. Existing technologies are unable to measure subcellular transcriptomics within an entire tissue sample in three dimensions at subcellular resolution. Furthermore, most transcriptomics methods require decoding steps that have to be customized to address the specific distortions and aberrations of each technology or microscope, making standardization and optimization difficult [88,89].

## 8. Computational Tools for Multi-omics Data Integration

With the advancement of technologies, such as genomics and transcriptomics based on second-generation sequencing, along with proteomics and metabolomics based on high-throughput MS, a large amount of complex and disordered data has been generated. It is necessary to employ bioinformatics analysis tools to integrate the intricate information. Compared with analyzing individual datasets separately, which is known as single-omics analysis, integrating and correlating various datasets is more conducive to uncovering the potential mechanisms of biological regulation and function, thereby enabling a comprehensive awareness of biological life processes. Integrating the sequencing and MS data obtained from single-omics analysis is the core of multi-omics research. The integration platform based on these conducts a cross-correlation analysis of the sequencing and the MS data and serves as the core platform for multi-omics analysis. Typical multi-omics integration analysis platforms (software packages) should meet three criteria: (1) process multi-omics data in parallel rather than sequentially; (2) be capable of integrating and analyzing data from at least two or more omics; and (3) have no specific requirements for the data format [90].

Examples include mixOmics, which provides a series of statistical methods for exploring and integrating multi-omics datasets, including traditional and regularized multivariate approaches; MetaboAnalyst, a comprehensive web-based platform for classifying, diagnosing, and integrating metabolomic and transcriptomic data; Omics Integrator, which is used to integrate data from different omics studies and identify significant molecular networks; and path view, which maps genomic data onto biological pathway diagrams and cooperates with multi-omics data analysis. Other examples involve multiOmicsViz for comparing and visualizing the relationships among multi-omics datasets; Ingenuity Pathway Analysis for uncovering, visualizing, and exploring associations and networks among multi-omics data; Joint and Individual Variation Explained for detecting shared and unique variations among multi-omics datasets; and Cytoscape for visualizing molecular interaction networks and integrating multi-omics data.

Currently, there is an increasing number of public-platform resources that meet the standards of multi-omics integration analysis data platforms, and there are also some publicly available databases, such as those listed in Table 1 and Table 2, which provide multi-omics datasets for patients. For instance, the GO database classifies gene functions into three main aspects: cellular component (CC), molecular function (MF), and biological process (BP). By making use of the GO database, we can determine the main associations of our target genes at the CC, MF, and BP levels. The KEGG database, along with similar ones like the WikiPathway and Reactome pathway databases, annotates gene functions and their involvements in various pathways in the human body. The Cancer Genome Atlas is one of the largest multi-omics databases, which contains clinical data, genomic variations, mRNA expression, miRNA expression, methylation, and other data for various human cancers, including subtypes of tumors. The Spatial Omics Database provides data from 26 spatial omics techniques, with a dataset size exceeding 50 million cells (spots). In addition to the specialized databases mentioned above for multi-omics, we also present other databases and their URLs in Table 1 and Table 2.

## 9. Future Perspective

### 9.1. Challenges

#### 9.1.1. The Complexity of Data Integration and Analysis

As the cost of sequencing decreases, the complexity of sequencing data increases. Dealing with large-scale datasets remains one of the greatest challenges in current multi-omics data analysis. Integrating and analyzing such data require highly specialized techniques and methods. Unlike single-omics analysis, multi-omics approaches focus on finding consistencies and correlations across different omics datasets to establish causal relationships within vast datasets. This demands significant computational resources to effectively integrate and interpret sequencing data from various technologies and platforms, along with advanced statistical and computational methods and extended processing times.

#### 9.1.2. Data Consistency and Standardization

Inconsistent data storage and formats across different technological platforms make data processing more challenging. There is a need for a unified framework that can handle and analyze multi-omics data in a simple, clear, and visual manner from start to finish. Developing new computational tools and algorithms to optimize data storage, retrieval, and analysis processes is essential for promoting industry standardization. This includes standardizing data formats and analytical tools, which is an urgent issue that needs to be addressed.

#### 9.1.3. The Rising Cost of Data Analysis

Despite the decrease in basic sequencing costs, the amount of sequencing required for multi-omics analysis is still significantly higher than for single omics. This increase in data volume demands more computational resources, which in turn, consumes more manpower and financial resources. The increase in data, along with the need for long-term storage, also significantly raises storage costs. Managing and storing large-scale data has become more complex and costly. These high costs limit the widespread application of multi-omics technologies. Therefore, it is crucial for researchers to select appropriate combinations of omics methods to avoid data waste and reduce analysis costs.

### 9.2. Prospects for Technology Application

Despite these challenges, multi-omics technologies have great potential in the field of drug-target identification. These integrated techniques have significantly impacted molecular-level drug discovery and development, playing a vital role in understanding disease mechanisms and identifying potential drug targets. In the coming decades, innovations in multi-omics technologies will further enhance our understanding of cell biology.

We can expect progress in several areas, including increased throughput, the establishment of standardized analysis formats, reduced costs for data analysis and storage, and the inclusion of more models in single assays. Additionally, improvements in the sensitivity and specificity of detecting and characterizing various forms of multi-omics measurements are anticipated.

### 9.3. Technological Advances and Breakthroughs

#### 9.3.1. Single-Cell Multi-omics and Spatial Multi-omics

With continuous improvements and breakthroughs in bioinformatics analysis and tool applications, the potential of multi-omics technologies for drug-target identification is expanding. New technologies and analytical methods, such as single-cell omics and spatial omics, enable us to visualize the molecular structure and functional phenotypes of tumors at single-cell resolution. This provides unique insights into the dynamic changes in tumor molecular structures during progression and treatment.

Future developments may see spatial omics evolving into single-cell spatial multi-omics, three-dimensional spatial omics, and spatiotemporal multi-omics. These advancements will offer more detailed and high-resolution data, further improving the accuracy and efficiency of drug-target identification. However, the application of these technologies in drug-target identification is still in its early stages and requires further validation and optimization.

#### 9.3.2. Multi-omics and Machine Learning

With the advancement of next-generation sequencing technologies, the cost of sequencing has gradually decreased, resulting in an explosion of sequencing data. Analyzing this vast amount of data manually is both time-consuming and labor-intensive. Artificial intelligence plays a crucial role in handling this data. Machine learning accelerates data processing and pattern recognition, significantly reducing the complexity of multi-omics data. This helps researchers extract valuable biological information from intricate datasets, enabling the rapid and efficient identification of potential therapeutic targets and offering smarter solutions for drug development.

## 10. Conclusions

In essence, multi-omics technology undeniably exhibits tremendous potential and promising prospects in the field of drug-target identification. A comprehensive and meticulous analysis of the data from multiple aspects through diverse means and approaches enables us to understand the pathogenesis of diseases in a more profound, intricate, and in-depth fashion. Significantly, this not only significantly accelerates the drug development process but also facilitates the realization of personalized medicine, which is of cardinal importance for tailoring treatment plans according to the unique and specific needs of individual patients.

Furthermore, with the continuous innovation and remarkable progress of sequencing technology, multi-omics technology is bound to progressively evolve into an absolutely indispensable and crucial tool in future drug development. It will not only tenaciously uphold its core and key role but also continuously broaden its application purview and enhance its influence, making progressively more substantial contributions to human health and medical progress. It will actively carve out new paths and generate new opportunities in the arduous battle against various diseases and the resolute pursuit of enhancing medical care outcomes, bringing cherished hope and practical solutions to multitudes of patients and a plethora of medical professionals. Through the seamless integration of various disciplines and technologies, multi-omics technology will continue to vigorously drive revolutionary and profound alterations in the medical field, ushering in a new era of more efficacious and personalized medical care.

## Figures and Tables

**Table 1:** A survey of common single-omics database information—including brief descriptions of each database (All websites were accessed on 11 June 2024).

Omic Name	Classification	Common Reference Databases	Website	Main Functions	Reference
Genomics	Three major DNA Databases	GenBank	http://www.ncbi.nlm.nih.gov/genbank/	Provides genome-wide 2D gel-electrophoresis profiles, collecting 2D gel-electrophoresis maps of proteomes of organisms with known genomic information.	[91]
		EMBL	https://www.ebi.ac.uk	Nucleic acid sequences, genomes, microarray gene expression, protein sequences, annotations, and many other biological data	[92]
		DDBJ	https://www.ddbj.nig.ac.jp/index-e.html	Genomic, transcriptomic, epigenomic, exomic, macrogenomic, macrotranscriptomic, and other multi-omics data for human, animal, and other samples.	[93,94]
Transcriptomics	NCBI	miRBase	http://www.mirbase.org	The most comprehensive miRNA database with nearly 40,000 miRNAs from more than 200 species.	[95,96]
	EMBL-EBI	LncRNAwiki	https://ngdc.cncb.ac.cn/lncrnawiki1/index.php/Main_Page	Integration of more than 100,000 LncRNAs currently available, classification of long non-coding RNAs	[97]
	NGDC	Rfam	http://rfam.org	Identification of non-coding RNAs, commonly used to annotate new nucleic acid sequences or genome sequences	[98,99]
Proteomics	Protein Sequence Database	UniProt	http://www.uniprot.org	Contains protein sequences, functional information, and an index of research papers.	[100]
		PIR	https://proteininformationresource.org	Database integrating public resources on protein function prediction data	[87]
		InterPro	http://www.ebi.ac.uk/interpro/	Integrated protein structural domain and functional site databases with data resources on protein families, domains, repeat sequences, and sites of action	[101,102]
	Protein Structure Database	Protein Data Bank	https://www.rcsb.org	X-ray diffraction structures, NMR spectra, electron microscopy (EM) imaging, and some special structures (e.g., DNA and RNA structure libraries) are included.	[103]
		SCOP	https://www.ebi.ac.uk/pdbe/scop/	Classifies known protein structures and describes the functions and evolutionary relationships of proteins of known structure based on the amino acid composition of different proteins and similarities in tertiary structure	[104]
	Proteome Database	PRIDE	https://www.ebi.ac.uk/pride/archive/	Classifies known protein structures and describes the functions and evolutionary relationships of proteins of known structure based on the amino acid composition of different proteins, as well as similarities in tertiary structure.	[105]
	Protein Functional Domain Database	PROSITE	https://prosite.expasy.org	Database of protein families and structural domains containing biologically significant sites, patterns, and statistical features that can help identify protein families	[106]
	Protein Interaction Database	DIP	https://dip.doe-mbi.ucla.edu/dip/Main.cgi	Tool for studying biological response mechanisms	[107]
Metabolomics	Metabolic Pathways Database	KEGG	https://www.kegg.jp	Linking genomic information to higher-order functional information for the study of genomes, metabolomes, signaling pathways, and biochemical reactions	[108,109]
		GO	https://geneontology.org	Standardizing the function of genes and proteins	[110]
	Metabolome commonly used Database	HMDB	https://hmdb.ca	Comprehensive reference information on human metabolites and their associated biological, physiological, and chemical properties	[111]

**Table 2:** Overview of common multi-omics databases characteristics (All websites were accessed on 11 June 2024).

Multi-omics Database	Website	Omics Assay Type	Reference
The Cancer Genome Atlas (TCGA)	https://www.cancer.gov/ccg/research/genome-sequencing/tcga	RNA-Seq, DNA-Seq, miRNA-Seq, SNV, CNV, DNA methylation, and RPPA	[112]
TARGET	https://www.cancer.gov/ccg/research/genome-sequencing/target	Gene expression, miRNA expression, copy number, and sequencing data	[113]
International Cancer Genomics Consortium (ICGC)	https://dcc.icgc.org/	Whole genome sequencing, genomic variation data (somatic and germline mutations)	[114]
OmicsDI	https://www.omicsdi.org	Proteomics, genomics, metabolomics, and transcriptomics data	[115]
METABRIC	https://www.cbioportal.org	Clinical features, gene expression, SNPs, and CNVs	[116]
CCLE	https://sites.broadinstitute.org/ccle	Gene expression, copy number, and sequencing data; pharmacological profile of 24 anticancer drugs	[117]
Roadmap Epigenomics	https://commonfund.nih.gov/epigenomics	RNA-seq, ChIP-seq (histones), DNase-seq, and methylation data	[118,119]

## Data Availability

No new data were created in this study. All the data reported in this review were found in original articles cited in the text.
